# Effectiveness of Treating Obstructive Sleep Apnea by Surgeries and Continuous Positive Airway Pressure: Evaluation Using Objective Sleep Parameters and Patient-Reported Outcomes

**DOI:** 10.3390/jcm13195748

**Published:** 2024-09-26

**Authors:** Yu-Ching Hsu, Jung-Der Wang, Sheng-Mao Chang, Ching-Ju Chiu, Yu-Wen Chien, Cheng-Yu Lin

**Affiliations:** 1 Department of Public Health, College of Medicine, National Cheng Kung University, Tainan 701, Taiwan; yuchinghsupro@gmail.com (Y.-C.H.); jdwang121@gmail.com (J.-D.W.); 2 Sleep Medicine Center, Tainan Hospital, Ministry of Health and Welfare, Tainan 700, Taiwan; 3 Department of Chinese Medicine, Tainan Hospital, Ministry of Health and Welfare, Tainan 700, Taiwan; 4 Department of Occupational and Environmental Medicine, National Cheng Kung University Hospital, College of Medicine, National Cheng Kung University, Tainan 701, Taiwan; 5 Department of Statistics, National Taipei University, Taipei 237, Taiwan; smchang110@mail.ntpu.edu.tw; 6 Institute of Gerontology, College of Medicine, National Cheng Kung University, Tainan 701, Taiwan; cjchiu@mail.ncku.edu.tw; 7 Department of Otolaryngology, National Cheng Kung University Hospital, College of Medicine, National Cheng Kung University, Tainan 704, Taiwan; 8 Sleep Medicine Center, National Cheng Kung University Hospital, College of Medicine, National Cheng Kung University, Tainan 704, Taiwan

**Keywords:** obstructive sleep apnea, long-term effects, patient-reported outcomes, mixed-effects model

## Abstract

**Background/Objectives**: Uvulopalatopharyngoplasty (UPPP), palatal plus nasal surgery (PNS), and continuous positive airway pressure (CPAP) are widely implemented treatments for obstructive sleep apnea (OSA). This study aims to explore the long-term effects on objective sleep parameters and patient-reported outcomes (PROs) following different therapeutic interventions for OSA. **Methods**: Data from patients with moderate-to-severe OSA were retrospectively collected from a medical center and a regional hospital, spanning from December 2011 to August 2018. Objective evaluations included the Apnea–Hypopnea Index (AHI), minimum O_2_ saturation, and sleep efficiency. The PROs consisted of the Snore Outcomes Survey and Epworth Sleepiness Scale. Using mixed-effects models, we evaluated longitudinal changes in sleep parameters and PROs, accounting for repeated measures and variations within individuals over time. **Results**: Among 448 patients with moderate-to-severe OSA, follow-up data were collected for 42 patients undergoing UPPP surgery, 171 undergoing PNS, 127 using CPAP, and 108 in the non-treated group. The mean follow-up was 16.7 months (SD = 11.9, range: 1.6–77.3). Significant improvements were observed in AHI, minimum O_2_ saturation, and hypersomnia immediately following interventions with UPPP, PNS, and CPAP therapy (*p* < 0.05). Moreover, the analysis revealed no significant rate of change in these parameters over time, suggesting that the benefits of these treatments were sustained in the long term. Furthermore, all interventions exhibited a significant short-term effect on self-reported snoring when compared to the control group, with a *p*-value of less than 0.001. However, the magnitude of this improvement gradually decreased over time. The snore scores seemed to return to pre-treatment levels among the UPPP, PNS, and CPAP groups after averages of 46.4, 63.5, and 74.4 months, respectively (all *p* < 0.05). **Conclusions**: Surgical interventions and CPAP therapy showed potential long-term effectiveness in managing OSA. Snoring symptoms reappeared about 3.9–5.3 years after surgical treatments, which seemed earlier than the average of 6.2 years in patients receiving CPAP and should be considered in patient-participatory decision-making processes.

## 1. Introduction

Obstructive sleep apnea (OSA) is a common sleep disorder characterized by recurrent episodes of a partial or complete obstruction of the upper airway during sleep, leading to frequent arousals and intermittent hypoxia. It is estimated that approximately 425 million adults globally suffer from moderate-to-severe OSA, highlighting its widespread prevalence [1,2]. The pathophysiology of OSA involves sympathetic activation, inflammation [3], and oxidative stress, which manifest as symptoms including snoring and daytime hypersomnia [2,3]. Additionally, OSA significantly increases the risk of cardiovascular diseases (CVDs), stroke, and all-cause mortality, making it a significant public health issue. Additionally, OSA significantly escalates the risk of cardiovascular diseases (CVDs), stroke, and all-cause mortality. A meta-analysis of prospective cohort studies revealed a dose–response relationship; each 10-unit increment in the Apnea–Hypopnea Index (AHI) is associated with a 17% increase in the risk of CVD [4]. These insights underscore the imperative for efficacious interventions [5] to ameliorate the physical and mental well-being of individuals, mitigate associated health consequences, and curtail healthcare expenditures [6].

The standard treatments for OSA include continuous positive airway pressure (CPAP) [7,8] and surgical interventions [9,10]. While several studies have assessed these treatments, they typically employed pre- and post-intervention controlled designs with follow-up durations ranging from 3 to 6 months [11,12,13]. Furthermore, only a limited number of studies extend their follow-up to 8 and 10 years, involving small cohorts of just 47 [10] and 17 [14] subjects, respectively, suggesting a slight decline in therapeutic effects over time. These studies predominantly focused on the AHI and daytime hypersomnia [11,12,13,15]; however, other critical indicators such as minimum O_2_ saturation, sleep efficiency, and self-reported snoring have not been extensively studied for long-term outcomes.

Randomized controlled trials (RCTs), deemed the gold standard for evaluating treatment efficacy, frequently impose stringent inclusion and exclusion criteria. These restrictions may not adequately capture the diversity among individuals with OSA [3,16,17,18], thereby potentially limiting the generalizability of their findings. Moreover, while healthcare systems in the United States [19,20], Canada [21], the United Kingdom [22], and Japan [23] provide coverage for CPAP and surgical interventions, Taiwan’s National Health Insurance (NHI) system currently reimburses sleep surgeries for OSA but does not cover CPAP. Generating real-world evidence that elucidates the long-term efficacy of different treatment modalities is vital to promote health equity in developing personalized management strategies [24,25]. Additionally, this kind of evidence is associated with enhanced self-management and increased patient satisfaction [26]. Therefore, this retrospective cohort study aimed to investigate both the short- and long-term impacts of surgical and CPAP treatments on objective sleep metrics and patient-reported outcomes (PROs) in individuals with moderate or severe OSA in Taiwan. We hypothesized that both surgical and CPAP interventions would yield sustained improvements in sleep quality metrics and PROs, contributing significantly to the management of OSA.

## 2. Materials and Methods

### 2.1. Study Population and Study Design

This retrospective study analyzed data from 3220 patients clinically suspected of having OSA, all of whom underwent polysomnography (PSG). These individuals were aged 20 years or older and their data were collected between December 2011 and August 2018 from the sleep laboratories of National Cheng Kung University Hospital (NCKUH) and Tainan Hospital of the Ministry of Health and Welfare. We enrolled patients diagnosed with moderate-to-severe OSA, defined by an initial AHI exceeding 15 events per hour. The exclusion criteria included the following: (1) PSG recordings for CPAP ventilation titration (because these recordings were primarily used to determine the initial CPAP settings and not for ongoing treatment evaluation); (2) patients who underwent nasal surgery, bariatric surgery, mandibular advancement surgery, and mandibular advancement orthosis; (3) patients with an initial Apnea–Hypopnea Index (AHI) of less than 15 events per h; and (4) patients with a single PSG data point only. Each included participant underwent PSG at least twice during the study period. The first PSG, conducted prior to any intervention, established a baseline, while subsequent PSGs assessed the effects of interventions or the progression in the no-intervention group. These assessments provided the necessary data to accurately calculate follow-up durations. Clinicians adhered to the Stanford sleep surgery protocol [27,28] and made surgical recommendations on the basis of endoscopic evaluations of upper airway obstruction [29]. UPPP surgery involves tissue rearrangement to increase the size of the airway, whereas palatal surgery focuses on reconstructing the airway, restoring airflow, and rehabilitating muscles [30]. PNS was recommended for patients with palatal and nasal blockage. Participants who declined surgery and received CPAP therapy and those who used CPAP regularly were assigned to the at-home CPAP group. Participants in the at-home CPAP group refrained from using CPAP during PSG recordings. This was carried out because PSG conducted with CPAP in use naturally yields good results, but many patients experience discomfort and poor compliance, leading to inconsistent usage. Therefore, our approach aimed to capture the lasting effects of CPAP on sleep quality and symptoms, even in the absence of immediate use during the study nights. Participants who had at least two PSG recordings but did not receive any treatment comprised the control group. This study included three treatment groups: UPPP, PNS, and at-home CPAP use. The control group received no treatment. Additionally, patients who switched their treatment modality, whether from surgery to CPAP or vice versa, during the follow-up period were excluded to ensure an accurate assessment of the longitudinal effects of each specific treatment. This study followed the STROBE guidelines for transparent reporting.

### 2.2. Outcome Measurement

Each patient was thoroughly evaluated for all objective and subjective sleep parameters at each assessment, encompassing both pre-intervention and all follow-up sessions.

#### 2.2.1. Objective Sleep Parameters

Objective sleep examinations were performed on all study participants by using standardized PSG instruments (Compumedics, Victoria, Australia) at both sleep centers. Trained technicians applied the 2007 American Academy of Sleep Medicine (AASM) scoring criteria from 2011 to 2014 and the 2014 AASM scoring criteria during the period of 2015–2018 to measure AHI, minimum oxygen saturation (minimum O_2_), and sleep efficiency [31]. AHI quantifies the number of respiratory events per h experienced by patients during sleep. Minimum O_2_ represents the lowest recorded level of O_2_ experienced by the patient. Sleep efficiency is calculated as the percentage of total sleep time spent in bed.

#### 2.2.2. Subjective Sleep Parameters (PROs)

PROs were assessed using two sleep questionnaires: the Snore Outcomes Survey (SOS) and the Epworth Sleepiness Scale (ESS). The SOS [32,33] consists of eight items assessing the duration, severity, frequency, and impact of self-reported snoring, where lower scores indicate more severe snoring symptoms, with scores ranging from 0 (worst) to 100 (best). ESS, which is an eight-item scale, measures daytime hypersomnia and yields a total score ranging from 0 (best) to 24 (worst).

#### 2.2.3. Covariates

Several potential confounders were assessed at each PSG evaluation to control for their influence on treatment outcomes. These included age, gender, and body mass index (BMI), as well as prevalent comorbidities such as hypertension, diabetes, myocardial infarction, and gastroesophageal reflux disease. The weight status of participants was categorized into three subgroups on the basis of their BMI, namely, BMI < 25, 25 ≤ BMI < 30, and BMI ≥ 30, following the classification previously described by others [34].

#### 2.2.4. Follow-Up Time

In this retrospective cohort study, we aim to evaluate the long-term efficacy of different treatments for OSA by analyzing changes in key sleep parameters over multiple follow-up periods. The term “follow-up time” refers to the duration during which each case was under observation. For example, the “follow-up time” is considered zero when a patient undergoes their first PSG. When the same patient has a second PSG, labeled as post-intervention follow-up 1, the “follow-up time” is calculated by subtracting the date of the first PSG from the date of the second PSG (post-intervention follow-up 1). Hence, each case, regardless of whether treatment was received, possesses at least one “follow-up time”, depending on the number of PSGs the patient underwent. This method ensures that every participant’s data contribute to our understanding of treatment efficacy over time, regardless of the number of PSGs they have undergone.

### 2.3. Statistical Analysis

The baseline sociodemographic characteristics, objective evaluations, and subjective evaluations across the four treatment types were summarized as frequencies (percentages) for categorical variables and means (standard deviations, SDs) for continuous variables. Differences in baseline characteristics among treatment groups were assessed using ANOVA. To further identify which specific treatment groups exhibited significant differences, post hoc pairwise comparisons were performed using Tukey–Kramer tests.

To rigorously assess the longitudinal effects of treatments on objective sleep parameters and PROs, we employed a mixed-effects model. This model was particularly suited for our study as it efficiently handled data across multiple time points and accommodated instances of missing measurements, a common challenge in longitudinal health research. Age, sex, and BMI, identified as significant predictors of moderate-to-severe OSA in a previous study, were included as covariates. Comorbidities associated with the treatment groups were entered into the model using a conservative *p*-value threshold of *p* < 0.15 to control for confounding [35]. Treatment groups, BMI, comorbidities, and “follow-up time” were treated as time-varying variables. To accurately evaluate the short- and long-term impacts of treatments for obstructive sleep apnea, our methodology was designed with specific analytical approaches. The coefficient for each treatment group signified its short-term impact, capturing immediate improvements right after treatment due to anatomical changes. The coefficient for “follow-up time” represented the natural change in outcome variables over time, which may occur as individuals age. In addition, an interaction term was created between each treatment group and the time post-treatment to assess whether the treatment effect could persist in the long term. If this interaction term was statistically significant, it indicated that the specific intervention group experienced a different rate of change in the outcome variable compared with the no-treatment group. The coefficient of the interaction term reflected the difference in the rate of outcome change per month during the entire follow-up period, which was defined as the long-term effect of the specific treatment. All statistical analyses were performed using SAS, with the level of statistical significance set at 0.05 (version 9.4, SAS Institute, Inc., Cary, NC, USA).

## 3. Results

### 3.1. Study Cohort

**Figure 1:**
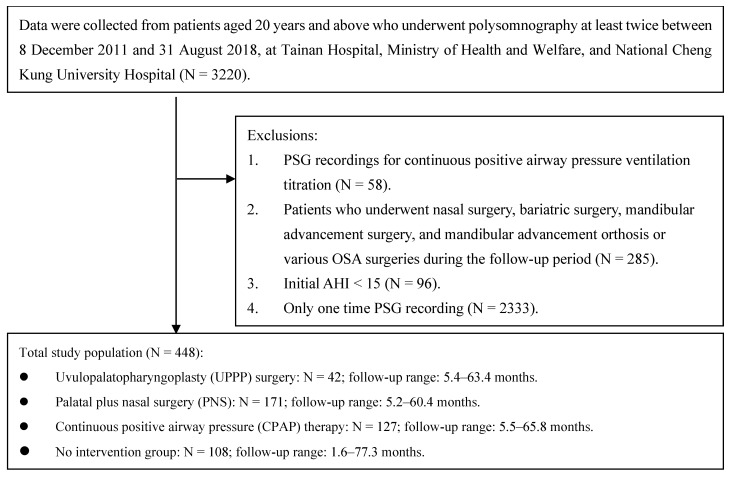
Flow diagram. Subject inclusion flow diagram.

A total of 448 patients with moderate-to-severe OSA aged 20 years or older and who had undergone PSG at least two times were included in the retrospective analysis (Figure 1 shows the flow diagram illustrating participant selection). Among them, 42 patients (9.4%) underwent UPPP surgery, 171 patients (38.2%) underwent PNS, 127 patients (28.3%) used CPAP at home, and 108 controls (24.1%) received no treatment. Table 1 summarizes the pre-treatment characteristics across the four treatment groups. Significant differences among groups were found for age (*p* < 0.001), sex (*p* = 0.003), BMI (*p* < 0.001), and comorbidities including hypertension (*p* = 0.002) and diabetes (*p* = 0.001). The CPAP group had the highest mean age, followed by the no-treatment, PNS, and UPPP surgery groups. The percentage of females was higher in the no-treatment group, and the CPAP group had a higher proportion of patients with obesity. Regarding objective evaluations, CPAP users had the highest baseline AHI and the lowest minimum O_2_ saturation. Among the surgical treatment groups, those who received UPPP surgery had poorer baseline objective evaluations than those who received PNS. Regarding PROs, self-reported snoring was less severe in the no-treatment group. No significant differences in hypersomnia were observed at baseline among the different treatment groups (*p* > 0.394). The mean follow-up was 16.7 months (SD = 11.9, range: 1.6–77.3). Of the 448 participants, 102 (22.7%) contributed more than two PSG records. The distribution of ‘follow-up time’ for each treatment group is detailed in Supplementary Table S1.

### 3.2. Mixed-Effect Models

The results of the mixed-effect model, delineating the short- and long-term effects of the three treatments compared with those of no treatment, are summarized in Table 2. With regard to objective sleep parameters, patients who underwent UPPP surgery exhibited a significant decrease in AHI (β = −14.59, 95% CI = from −22.36 to −6.82) and an improvement in sleep efficiency (β = 5.98, 95% CI = from 1.28 to 10.69) post-surgery compared with the no-treatment group. Similarly, patients who underwent PNS and those utilizing CPAP at home reported improved AHI (β **=** −5.98, 95% CI = from −9.56 to −2.40; β = −4.74, 95% CI = from −8.69 to −0.79) and minimum O_2_ saturation (β = 3.38, 95% CI = from 1.66 to 5.09; β = 3.96, 95% CI = from 2.08 to 5.85) after treatment. Regarding the long-term effects on objective sleep parameters, the coefficients for the “follow-up time” and the interaction terms between the treatment group and the time post-treatment for AHI, sleep efficiency, and minimum O_2_ saturation were not statistically significant. This finding suggests that the improvement in these outcomes after surgeries and CPAP could persist during the follow-up period.

Upon evaluating the short-term effect of each treatment on PROs, as documented in Table 2, a significant short-term improvement in PROs was observed after patients received any of the treatments, compared with those who had no intervention. The effects on self-reported snoring decreased over time during the follow-up, as indicated by the statistically significant coefficient for the “follow-up time” variable. The participants exhibited an average improvement of 0.23 scores per month (β = 0.23, 95% CI = from 0.11 to 0.34) in self-reported snoring, irrespective of whether treatment was administered or not. Although all three treatment groups exhibited significant short-term improvements in self-reported snoring scores, these improvements did not appear to be sustainable, as indicated by the significant interaction terms. These terms revealed that the self-reported snoring scores deteriorated at a higher rate over time after treatments than that of the no-treatment group. For instance, the PNS group seemed to have a short-term improvement in self-reported snoring scores by 22.23 (95% CI = from 19.05 to 25.40) post-surgery. However, after surgery, the score declined at an average rate of 0.35 {0.23 [95% CI = from 0.11 to 0.34] + [−0.58 (95% CI = from −0.82 to −0.34)]} per month, leading the self-reported snoring scores to revert to pre-surgery levels after approximately 63.5 (22.23/0.35) months. Therefore, despite initial improvements in self-reported snoring scores being observed for these treatments, these scores returned to pre-treatment levels among the UPPP, PNS, and CPAP groups after averages of 46.4, 63.5, and 74.4 months, respectively. Notably, the CPAP group exhibited the longest duration before the snoring scores rebounded to baseline levels.

## 4. Discussion

To the best of the authors’ knowledge, this retrospective study, utilizing mixed-effect models, represents the first scholarly investigation into the objective and subjective effects of three OSA treatments—UPPP, PNS, and CPAP—with adjustments for potential confounders. Adult patients with moderate-to-severe OSA receiving appropriate treatment modalities, including sleep surgeries and CPAP, showed significant improvements in objective sleep parameters and hypersomnia over time compared with the no-treatment group. This observation reinforces the rationale for investing in the development of personalized management strategies for OSA. Although surgery and CPAP showed potential long-term effectiveness, patients must be informed that snoring seems to reappear earlier after surgery in patient-participatory decision-making. Future studies are warranted for the long-term follow-up of these patients to observe the persistent effectiveness and benefits.

Previous studies emphasize the necessity of regular, ongoing follow-ups to monitor symptom resolution in OSA interventions [36]. In surgical treatments, the literature suggests that OSA interventions, particularly UPPP, yield short-term therapeutic benefits on objective sleep parameters [11,12,13]. Although limited follow-up studies in randomized controlled trials (RCTs) with small sample sizes (29 and 47 subjects) indicate a reduction in therapeutic effects over extended periods, ranging from 3 to 8 years [37,38,39], the present study demonstrated sustained effects within its shorter follow-up timeframe. This observed discrepancy may be partially attributed to our study design, which employed a mixed-effects model to account for potential covariates such as age and BMI. Despite a shorter follow-up duration, this study encompassed a comprehensive sample, thereby achieving a robust treatment effect with broader applicability than RCTs, which often exclude subjects with extreme BMI or other outcome-related comorbidities [16]. Furthermore, the present study specifically showed that patients undergoing UPPP and PNS experienced reductions in AHI by 14.59 and 5.98 events/h, respectively. According to Wang et al., these reductions are potentially associated with 24.8% and 10.17% decreases in CVD risk, respectively [4].

Limited research has investigated the trajectory of sleep efficiency post-treatment. The findings of this study suggest that UPPP is the only intervention associated with improvement during the follow-up period. A previous study indicated a slight initial improvement during the follow-up [38]. This consistent therapeutic benefit may be linked to more pronounced anatomical enhancements at the site of obstruction than PNS [40].

For PROs’ effect after receiving surgical treatments, the analysis indicated a sustained improvement in hypersomnia. A previous systematic review highlighted an improvement in ESS when comparing pre- and post-treatment scores [41]. Furthermore, other 3- and 8-year follow-up studies demonstrated persistent improvement in ESS [38,39]. These observations are consistent with the findings of the present study, indicating a potential for long-term improvement in hypersomnia through these treatments.

The findings on subjective-reported snoring are in agreement with those of prior research indicating that the initial benefits may not be sustained long-term. Studies have recorded improvements in snoring within 2–6 months postoperatively [42,43] and observed a recurrence at 31.3 months after UPPP [44]. Moreover, UPPP can temporarily remove factors, such as enlarged adenoids and tonsils; a long uvula; a redundant soft palate; and poor muscle tone in the pharynx, palate, and tongue [45]. However, the benefits of this pharyngeal enlargement may decrease over time, potentially leading to the return of snoring symptoms [46]. Additionally, the participants exhibited an average monthly improvement of 0.23 points (β = 0.23, 95% CI = from 0.11 to 0.34) in self-reported snoring, regardless of treatment administration. This observation could be attributed to individuals becoming habituated to their snoring, subsequently reporting diminished concern over time. The present study suggests a potential reduction in the efficacy of surgical treatments approximately 46–64 months post-operation.

In this cohort, regular CPAP users underwent routine follow-up care but did not use their CPAP during PSG recordings. This practice was followed because, prior to 5 April 2017, Taiwan’s policy allowed individuals with an AHI exceeding 40 events per hour to qualify for a disability certificate, which provided financial benefits such as reduced co-payments and tax reductions. Despite the non-use of CPAP on the night of PSG recordings, this study showed a residual improvement in AHI of 4.74 events/h, a 3.96% increase in minimum O_2_ saturation, and a 1.29-point reduction in ESS scores. These improvements likely resulted from transient reductions in snoring-related inflammation due to regular CPAP therapy before the PSG. Furthermore, the observed residual AHI reduction of 4.74 events/h may correspond to an 8.08% decrease in CVD risk [4], as noted by Wang et al. However, it is important to note that CPAP would likely have been even more effective had it been used during PSG recordings, as evidenced by a meta-analysis showing a reduction of 23 events/h in AHI with CPAP use [4], potentially leading to a 39.2% reduction in CVD risk [4]. However, CPAP adherence, influenced by factors like device noise, mask comfort, cost, and dry mouth, remains low at 34.1% [8]. Thus, identifying CPAP users who could achieve good outcomes and high adherence is critical.

In terms of long-term efficacy, our findings underscore the sustained improvements observed in AHI, minimum O_2_ saturation, and hypersomnia, supporting the inclusion of CPAP within Taiwan’s National Health Insurance (NHI) framework. It is crucial to consider the international variations in CPAP and OSA surgical treatment coverage when discussing the integration of CPAP into Taiwan’s NHI system. For instance, the national insurance programs of the healthcare systems in the United States [19,20] and the United Kingdom [22] subsidize a preliminary CPAP trial ranging from 3 months to 6 months for patients diagnosed with OSA; if effectiveness was found on AHI and hypersomnia, plus patients’ good compliance, the subsequent CPAP expenses covered by insurance may provide long-term health effects. In contrast, Taiwan’s NHI covers sleep surgeries for OSA but does not extend coverage to CPAP usage. Consequently, patients must bear out-of-pocket expenses ranging from approximately TWD 50,000 to TWD 160,000 (approximately USD 2000–5000). The CPAP users in this study, with a baseline mean AHI of 66.0 events/h, had more severe conditions than those who received surgeries, but were not covered by NHI, highlighting a substantial inequity in healthcare provision. Therefore, incorporating CPAP therapy into the NHI could enhance health equity for patients with severe OSA, especially those unsuitable for surgical interventions. Future health insurance policies should consider offering a trial period for CPAP use for patients with contraindications to sleep surgeries (e.g., older age, severe pulmonary diseases, and unstable cardiovascular status). The continuation of CPAP coverage should then be contingent upon demonstrating good compliance and improvements in AHI and hypersomnia during the trial.

This study also supports the use of treatments that address subjective and objective symptoms to aid in patient-participatory decision-making in managing OSA. The literature indicates that the goals for adult OSA treatment vary depending on age, symptoms, and the severity of the condition [47]. However, only one-third of adults with OSA engage in discussions with their healthcare providers about their treatment decisions [48]. Surgeries and CPAP could work if the main concerns in patients were about OSA severity and hypersomnia. Alternatively, if the patient was particularly troubled by snoring, the findings suggest that relief of the snoring symptom seemed to persist longer among CPAP users. This new observational evidence could be useful to the shared decision-making process in making a more suitable choice.

This study has several limitations. First, our study’s retrospective design introduces a potential selection bias [49], as patients who did not return for subsequent assessments were excluded. A comparison of characteristics between the “No Intervention” group and the “Excluded” group, delineated in Supplementary Table S2, reveals that the “No Intervention” group had lower BMI, suggesting a possible underestimation of the findings due to this sample characteristic. More critically, the small sample size of patients available for long-term follow-up assessments further complicates this issue, restricting our confidence in assessing the long-term efficacy of the treatments evaluated. Second, the changes in AASM scoring criteria between 2011 and 2014 may have influenced the measurement of the AHI values. The AASM 2014 criteria led to a median percentage increase of 77% in AHI compared with the AASM 2007 criteria [50]. Third, this study did not account for various behavioral attributes that could potentially influence the outcomes, including dietary habits, lifestyle factors, exercise routines, behavioral modifications, weight loss, smoking cessation, and the use of substances such as alcohol and tobacco. Fourth, quantifiable data on CPAP compliance were not available, and if actual CPAP compliance is lower than what was self-reported, the observed therapeutic effects might be more substantial than currently indicated. Therefore, it is recommended that future research includes detailed CPAP adherence data, which would allow for a more thorough assessment of how treatment adherence impacts clinical outcomes. Fifth, we did not explore the effects of mandibular advancement surgery and mandibular advancement orthosis in managing OSA. Given the high out-of-pocket costs in Taiwan—approximately USD 12,500 for surgery and USD 3000 for orthosis—and the infrequency of these interventions at the two participating hospitals, they were excluded from this study. Future studies should consider these interventions when assessing long-term treatment effects. Finally, the generalizability of the results is predominantly confined to an Asian demographic and to patients treated at two hospitals in Taiwan. Future research should encompass a broader range of healthcare facilities to elucidate the efficacy of OSA treatments across diverse clinical settings.

## 5. Conclusions

This real-world, repeated-measure follow-up study found significant and sustained improvements in AHI, minimum O_2_ saturation, and hypersomnia following sleep surgeries and CPAP as treatment outcomes for OSA. In Taiwan, the health insurance coverage should be broadened to encompass CPAP devices, and benefits should be extended to a widened spectrum of patients with OSA. Additionally, our results offer valuable insights for personalized decision-making. Notably, snoring symptoms tended to reoccur sooner following surgical interventions—typically within 3.9 to 5.3 years—compared to an average of 6.2 years for patients treated with CPAP. This differential in symptom recurrence underscores the importance of incorporating such data into patient-centered decision-making processes, allowing for more informed treatment choices based on expected long-term outcomes.

## Figures and Tables

**Table 1:** Baseline characteristics of participants among the four types of treatment groups.

	UPPP Surgery ^a^ (N = 42)	Palatal Plus Nasal Surgery (N = 171)	CPAP ^b^ (N = 127)	No Intervention (N = 108)	*p*-Value
Age (mean [SD]) ^†‡§¶#^	44.0 (9.7)	46.3 (11.3)	51.9 (12.2)	50.8 (13.4)	<0.001
Female (No. [%]) ^#^	9 (21)	29 (17)	12 (9)	30 (27)	0.003
BMI (mean [SD]) *^§#^	29.6 (5)	26.9 (3.8)	29.8 (5.5)	28 (5.2)	<0.001
BMI < 25 (No. [%]) ^§#^	9 (21)	51 (30)	24 (19)	33 (31)	<0.001
25 ≤ BMI < 30 (No. [%])	16 (38)	90 (53)	51 (40)	52 (48)	
BMI ≥ 30 (No. [%])	17 (41)	30 (18)	52 (41)	23 (21)	
Comorbidities					
Hypertension ^§^	18 (43)	58 (34)	71 (56)	46 (43)	0.002
Diabetes ^§^	4 (9)	7 (4)	23 (18)	13 (12)	0.001
Myocardial infarction	0 (0)	4 (2)	3 (2)	3 (3)	0.773
GERD ^f^	0 (0)	2 (1)	3 (2)	6 (6)	0.388
Objective evaluation					
AHI (per hour) ^c^ (mean [SD]) *^†§#^	53.1 (25.0)	39.1 (18.4)	66.0 (20.9)	44.0 (21.6)	<0.001
Minimum SpO_2_ (%, mean [SD]) ^†§#^	72.9 (11.3)	74.5 (11.5)	66.7 (13.5)	74.8 (11.7)	<0.001
Sleep efficiency (%, mean [SD])	82.0 (12.6)	85.6 (9.0)	82.2 (12.0)	82.1 (12.6)	0.019
Patient-reported outcomes					
Self-reported snoring (SOS ^d^ questionnaire, mean [SD]) ^¶#^	43.9 (13.2)	43.4 (13.7)	45 (14.3)	50.2 (15.0)	0.001
Hypersomnia (ESS ^e^ questionnaire, mean [SD])	9.9 (4.4)	10.6 (4.3)	10.8 (4.8)	9.9 (5.1)	0.394

^a^ UPPP: uvulopalatopharyngoplasty. ^b^ CPAP: continuous positive airway pressure. ^c^ AHI: Apnea–Hypopnea Index. ^d^ SOS: Snore Outcomes Survey. ^e^ ESS: Epworth Sleepiness Scale. ^f^ GERD: gastroesophageal reflux disease. Post hoc pairwise comparisons were conducted using Tukey–Kramer tests to identify significant differences between specific treatment groups. The symbols in the table represent the following significant differences at the 0.05 level. *: UPPP surgery group vs. palatal plus nasal surgery group. ^†^: UPPP surgery group vs. CPAP group. ^‡^: UPPP surgery group vs. no-intervention group. ^§^: Palatal plus nasal surgery group vs. CPAP group. ^¶^: Palatal plus nasal surgery group vs. no-intervention group. ^#^: CPAP group vs. no-intervention group.

**Table 2:** Coefficient estimates of objective evaluations and subjective sleep parameters in adults among different treatment groups.

	Objective Sleep Parameters			Subjective Sleep Parameters	
Fixed Effect ^a^	AHI ^d^	Minimum SpO_2_	Sleep Efficiency	Self-Reported Snoring (SOS ^e^ Questionnaire)	Hypersomnia (ESS ^f^ Questionnaire)
Treatment (ref: no treatment)					
UPPP surgery ^b^	−14.59 (−22.36, −6.82) ***	3.87 (0.12, 7.62) *	5.98 (1.28,10.69) *	25.97 (19.18, 32.75) ***	−3.53 (−5.32, −1.74) ***
Palatal plus nasal surgery	−5.98 (−9.56, −2.40) **	3.38 (1.66, 5.09) ***	1.61 (−0.58, 3.81)	22.23 (19.05, 25.40) ***	−1.62 (−2.44, −0.79) ***
CPAP ^c^ use at home	−4.74 (−8.69, −0.79) *	3.96 (2.08, 5.85) ***	0.15 (−2.28, 2.58)	13.39 (9.87, 16.92) ***	−1.29 (−2.20, −0.37) **
Follow-up time (per month)	0.02 (−0.11, 0.16)	0.06 (−0.001, 0.13)	−0.004(−0.08,0.00)	0.23 (0.11, 0.34) ***	−0.005 (−0.04, 0.03)
Interaction with post-treatment time ^g^ (per month)					
UPPP surgery	−0.03 (−0.76, 0.70)	0.01 (−0.34, 0.36)	−0.25 (−0.70, 0.19)	−0.79 (−1.42, −0.15) *	0.04 (−0.13, 0.21)
Palatal plus nasal surgery	−0.17 (−0.44, 0.11)	−0.10 (−0.23, 0.03)	−0.04 (−0.20, 0.13)	−0.58 (−0.82, −0.34) ***	0.03 (−0.04, 0.09)
CPAP use at home	0.12 (−0.10, 0.32)	−0.003 (−0.10, 0.10)	−0.10 (−0.23, 0.03)	−0.41 (−0.59, −0.22) ***	0.03 (−0.02, 0.08)

^a^ Age, sex, BMI, comorbidities, and follow-up time were controlled in this model. ***: *p* < 0.001; **: *p* < 0.01; *: *p* < 0.05. ^b^ UPPP: uvulopalatopharyngoplasty. ^c^ CPAP: continuous positive airway pressure. ^d^ AHI: Apnea–Hypopnea Index. ^e^ SOS: Snore Outcomes Survey. Higher scores indicate better effect. ^f^ ESS: Epworth Sleepiness Scale. Higher scores indicate worse effect. ^g^ Post-intervention time: the duration after each interaction (month).

## Data Availability

The data for this study are available to qualified researchers upon request from the first author, Yu-Ching Hsu.

## Supplementary Materials

The following supporting information can be downloaded at: https://www.mdpi.com/article/10.3390/jcm13195748/s1, Table S1. Distribution of follow-up time among four kinds of treatment groups; Table S2. Baseline characteristics of included and excluded groups.
