# Supplementary material for: Effectiveness of Treating Obstructive Sleep Apnea by Surgeries and Continuous Positive Airway Pressure: Evaluation Using Objective Sleep Parameters and Patient-Reported Outcomes
Source: J Clin Med. 2024 Sep 26;13(19):5748. doi: 10.3390/jcm13195748 (PMC11476387; doi:10.3390/jcm13195748)
Supplement: Supplementary file 1 [file jcm-13-05748-s001.zip › jcm-3175330-supplementary.pdf]

Supplementary Table S1. Distribution of follow-up time among four kinds of treatment groups.

	UPPP surgery [b] (N = 42)		Palatal plus Nasal surgery (N = 171)		CPAP [c] (N = 127)		No. of intervention (N = 108)	
	N	Month (SD)	N	Month (SD)	N	Month (SD)	N	Month (SD)
Pre-intervention evaluation [a]	42	-	171	-	128	-	108	-
Post-intervention follow-up 1	42	8.0 (6.6)	171	8.5 (6.1)	128	11.8 (7.8)	108	16.9 (12.1)
Post-intervention follow-up 2	1	23.4 (-)	23	24.1 (10.9)	43	26.1 (11.2)	8	37.9 (10.4)
Post-intervention follow-up 3			3	36.7 (18.5)	18	36.1 (11.1)		
Post-intervention follow-up 4			1	38.3 (-)	4	45.0 (12.3)		
Post-intervention follow-up 5					1	42.8 (-)		

[a] Month: the measured duration of each intervention from the first time to sixth time.

[b] UPPP: uvulopalatopharyngoplasty.

[c] CPAP: continuous positive airway pressure.

Supplementary Table S2. Baseline characteristics of included and excluded groups.

	**Surgery groups[a] (N = 213)**	**CPAP[b] (N = 127)**	**No intervention (N = 108)**	**Excluded groups[c] (N = 2,333)**	***P* value [d]**
**Age (mean [SD])**	45.9 (11)	51.9 (12.2)	50.8 (13.4)	49.1 (14.4)	0.226
**Female (No. [%])**	38 (18)	12 (10)	30 (28)	553 (20)	0.332
**BMI (mean [SD])**	27.4 (4.2)	29.8 (5.5)	28.0 (5.2)	29.4 (6.5)	0.026
**BMI < 25 (No. [%])**	60 (28)	24 (19)	33 (31)	557 (20)	0.004
**25 ≤ BMI < 30 (No. [%])**	106 (50)	51 (40)	52 (48)	934 (34)	
**BMI ≥ 30 (No. [%])**	47 (22)	52 (41)	23 (21)	841 (30)	
**Comorbidities**					
**Hypertension**	76 (36)	71 (56)	46 (43)	937 (40)	0.615
**Diabetes**	11 (5)	23 (18)	13 (12)	320 (14)	0.619
**Myocardial infarction**	4 (2)	3 (2)	3 (3)	57 (2)	0.826
**GERD[i]**	107 (50)	59 (46)	44 (41)	1216 (52)	0.021
**Objective evaluation**					
**AHI (per hour)[e]**	41.9 (20.6)	66.0 (20.9)	44.0 (21.6)	45.8 (24.4)	0.444
**Minimum SpO_2 (%)**	74.2 (11.4)	66.7 (13.5)	74.8 (11.7)	75.1 (11.7)	0.792
**Sleep efficiency (%)**	84.9 (9.9)	82.2 (12.0)	82.1 (12.6)	80.0 (13.6)	0.119
**Patient-reported outcomes**					
**Self-reported snoring (SOS[f] questionnaire)**	43.5 (13.6)	45.0 (14.3)	50.2 (15.0)	49.3 (14.8)	0.538
**Hypersomnia (ESS[g] questionnaire)**	10.4 (4.3)	10.8 (4.8)	9.9 (5.1)	9.2 (4.9)	0.163

[a] Surgery groups: Moderate-to-severe obstructive sleep apnea patients receiving UPPP (uvulopalatopharyngoplasty) or palatal plus nasal surgery.
[b] CPAP: Continuous positive airway pressure.
[c] Excluded groups: Patients with moderate-to-severe obstructive sleep apnea take sleep test once only.
[d] P-value: This metric was utilized solely to compare the characteristics between the "No Intervention" group and the "Excluded" group.
[e] AHI: Apnea-Hypopnea Index.
[f] SOS: Snore Outcomes Survey.
[g] ESS: Epworth Sleepiness Scale.
